# Refining seizure foci localization: the potential of TSPO-PET

**DOI:** 10.1186/s42494-025-00234-2

**Published:** 2025-08-21

**Authors:** Yiqiao Wang, Yuncan Chen, Shimin Xu, Xunyi Wu

**Affiliations:** 1 Department of Neurology, Huashan Hospital, Fudan University, Shanghai, 200040 China; 2 National Center for Neurological Disorders, 12 Wulumuqi Zhong Road, Shanghai, 200040 China

**Keywords:** Translocator protein, Positron emission tomography, Drug-resistant epilepsy, Seizure foci, Neuroinflammation

## Abstract

Translocator protein positron emission tomography (TSPO-PET) is a novel imaging modality that leverages the high expression of TSPO in activated microglia and other cells within seizure foci. It has been increasingly applied in the preoperative evaluation of drug-resistant epilepsy (DRE) to aid in the localization of these foci. With advances in tracer development, TSPO-PET has achieved higher signal-to-noise ratios and broader clinical utility. Clinical studies indicate that TSPO-PET yields significantly higher positive detection rates for seizure foci compared to magnetic resonance imaging and fluorodeoxyglucose positron emission tomography. This review summarizes recent progress in TSPO-PET radiotracer technology, its mechanism of action, and its clinical applications for managing DRE.

## Background

Epilepsy is a chronic neurological disorder characterized by recurrent seizures and an enduring predisposition to generate epileptic seizures [1]. Pharmacotherapy remains the first-line treatment for epilepsy, while nearly one-third of patients are classified as having drug-resistant epilepsy (DRE) after failing to achieve seizure control despite appropriate medical treatment [2]. Surgical intervention remains a viable option for some patients; however, its success critically depends on the precise localization of the seizure foci [3, 4]. Traditional imaging modalities, such as magnetic resonance imaging (MRI) and $^{18}$F-2-fluoro-deoxy-D-glucose positron emission tomography (FDG-PET), are often inadequate for detecting subtle structural or functional abnormalities associated with seizure foci [5]. This limitation underscores the need for innovative imaging techniques.

Translocator protein (TSPO), an 18-kDa protein located on the outer mitochondrial membrane, is a biomarker of neuroinflammation. In the central nervous system, TSPO expression is upregulated in activated microglia, reactive astrocytes, and other glial cells during pathological states [6], including epilepsy. TSPO’s pivotal role in neuroinflammation has spurred the development of TSPO-PET, a novel imaging approach that exploits radiolabeled TSPO ligands to visualize regions of neuroinflammatory activity [7].

TSPO-PET has emerged as a promising tool for localizing seizure foci, particularly in DRE cases where other imaging modalities fail. Recent advances in radiotracer design have significantly improved its sensitivity and specificity, facilitating the detection of inflammation-associated seizure foci [8, 9]. Additionally, TSPO-PET offers insights into the pathophysiological processes underlying epilepsy, including the interplay between neuroinflammation and neuronal hyperexcitability [10].

This article provides a comprehensive review of the mechanism underlying TSPO-PET, recent advances in radiotracer development, and the clinical applications of this technique in DRE management.

## Mechanism of TSPO-PET in localizing seizure foci

### The expression of TSPO in epilepsy

The expression and distribution of TSPO are markedly upregulated in pathological conditions compared to healthy states. Under physiological conditions, TSPO is expressed in limited brain regions, such as the olfactory bulb, choroid plexus, and ependyma, with minimal expression in most brain tissues [11, 12]. However, under certain pathological states like Alzheimer’s disease [13], multiple sclerosis [14] and stroke [15], TSPO expression significantly increases in affected regions.

In epilepsy, a consistent upregulation of TSPO has been observed in both patients and animal models. In brain tissue samples from patients with temporal lobe epilepsy (TLE), TSPO expression is significantly increased in the hippocampus, particularly in the sclerotic regions ipsilateral to the seizure focus [7, 16–20]. Immunohistochemical and transcriptomic analyses have demonstrated that this upregulation predominantly occurs in glial cells, including activated microglia and astrocytes, and correlates with neuropathological hallmarks such as gliosis and hippocampal sclerosis [7, 16–18]. These findings suggest that TSPO is enriched in seizure foci and is closely linked to chronic neuroinflammation in epilepsy.

In experimental models, such as kainic acid [21–24], and pilocarpine-induced epilepticus models [25], TSPO expression is significantly upregulated in glia cells [23, 25, 26], within the hippocampus, thalamus, hypothalamus, amygdala and cortex [23, 27]. The upregulation occurs rapidly after seizure induction, peaks within 2 weeks, and may persist for weeks or months, indicating a prolonged inflammatory response [23, 28].

The upregulation of TSPO in epileptic brains is believed to be driven by neuroinflammatory processes. Seizure activity leads to neuronal injury and the release of damage-associated molecular patterns (DAMPs), which trigger activation of microglia and astrocytes [29]. This glial activation contributes to elevated TSPO expression, reflecting the extent and persistence of inflammation [30]. Given its strong association with glial activation and tissue damage, TSPO serves as a reliable indicator of neuroinflammation and may represent a promising biomarker in epilepsy.

### Biological mechanisms of TSPO in neuroinflammation

TSPO has been implicated in a variety of cellular processes, including steroidogenesis, heme synthesis, mitochondrial energy metabolism, apoptosis, and autophagy [31]. These functions are relevant to how cells respond to stress and inflammation, and thus may underlie TSPO’s upregulation in neuroinflammatory conditions. However, direct evidence linking these biological pathways to epilepsy remains limited. In this section, we summarize the potential mechanisms through which TSPO may regulate neuroinflammation, as suggested by studies in other neurological disorders and in vitro models. These pathways, while not specific to epilepsy, may help explain TSPO’s role in epileptic neuroinflammation and highlight its potential as a therapeutic target.

#### Steroidogenesis

One of the most distinctive features of TSPO is its role in steroidogenesis. TSPO is thought to facilitate the transport of cholesterol into mitochondria through a conserved cholesterol recognition/interaction amino acid consensus (CRAC) motif [32], enabling the synthesis of neurosteroids. While some studies support this mechanism [33–35], others have challenged TSPO’s necessity in steroidogenesis, particularly in microglia, using TSPO knockout models [36, 37]. Given the established role of neurosteroids in modulating inflammation [38], the involvement of TSPO in neurosteroidogenesis suggests a potential link between TSPO expression and neuroinflammatory responses in epilepsy.

#### Heme synthesis

TSPO also participates in mitochondrial heme transport and porphyrin handling [32]. It is thought to bind heme via a WYXXLXKP motif and facilitate its export via another conserved motif [39]. The processed heme can support gp91phox assembly and promote NADPH oxidase activity, which activates Nrf2-mediated transcription of antioxidant genes [40]. These responses are believed to counteract oxidative stress during neuroinflammatory processes.

Loss of TSPO has been associated with porphyrin accumulation and increased mitochondrial reactive oxygen species (ROS) generation [41]. Because dysregulated heme metabolism has been linked to ferroptosis, a lipid peroxidation-driven and inflammation-associated form of cell death [42, 43], TSPO’s involvement in this pathway may represent an additional mechanism contributing to neuroimmune regulation. In the context of epilepsy, such mechanisms could influence the extent of oxidative damage and inflammation, although direct evidence remains lacking.

#### Mitochondrial function and mitophagy

TSPO is closely associated with the mitochondrial permeability transition pore (mPTP) [44], a complex involving voltage-dependent anion channel 1 (VDAC1) [45, 46]. Although TSPO is not essential for mPTP formation [47], its ligands can modulate pore activity, thereby influencing mitochondrial permeability, cell survival, and apoptosis [48, 49].

TSPO has also been implicated in regulating mitophagy—the selective clearance of damaged mitochondria—which is essential for controlling ROS and preventing neuroinflammation [50]. TSPO overexpression can disrupt mitophagy by interfering with VDAC1 ubiquitination and SQSTM1 recruitment, leading to impaired mitochondrial clearance and increased oxidative stress [51, 52]. Conversely, certain TSPO ligands may restore mitophagy by enhancing SQSTM1 expression [53, 54].

These findings highlight a dual role for TSPO in mitochondrial regulation and support its involvement in neuroinflammatory responses. Although direct evidence in epilepsy models remains limited, these mitochondrial mechanisms may underlie the association between TSPO upregulation and epilepsy pathology.

### Microglia is related to the neuroinflammation and epilepsy

Microglia, the resident immune cells of the central nervous system, play a crucial role in neuroinflammation and are significantly involved in the pathogenesis of epilepsy. Microglia exist in three states: resting (M0), pro-inflammatory (M1), and anti-inflammatory (M2). In seizure foci, most activated microglia differentiate into the M1 phenotype [55], which are in response to seizure insults, such as status epilepticus, leading to the production of pro-inflammatory cytokines [56] and chemokines [57]. Neuroinflammation subsequently reactivates microglia, exacerbating epilepsy pathologies and creating a vicious cycle [58]. Given that activated microglia are major sources of TSPO expression in the brain, their spatiotemporal dynamics may underlie the increased TSPO-PET signal observed in seizure regions, which will be further discussed in the next section.

### Mechanism of increased tracer uptake in seizure foci

Traditionally, the increased tracer uptake observed in TSPO-PET was attributed to the overexpression of TSPO in activated microglia within seizure foci. While TSPO can be induced in various cell types, activated microglia are considered the principal source in this context. Mechanistically, TSPO expression can be upregulated upon inflammatory or seizure stimuli through a defined signaling cascade involving protein kinase C epsilon (PKC$\epsilon$). This kinase activates the MAPK pathway (Raf-1-MEK1/2-ERK1/2), which in turn promotes the phosphorylation and activation of two key transcription factors: STAT3 and c-Jun. These transcription factors directly bind to the TSPO promoter and enhance its transcription. Altogether, this cascade exemplifies a tightly regulated transcriptional mechanism by which extracellular stimuli like inflammation or seizures are transduced via PKC$\epsilon$-MAPK signaling to upregulate TSPO expression [59].

To link this regulatory mechanism to specific cell types, several studies have examined TSPO expression in glial cells within seizure regions. Amhaoul et al. [28] quantified the relationship between TSPO expression and microglial and astrocytic activation, demonstrating that TSPO expression is tightly correlated with microglial activity but only weakly associated with astrocytes. Similarly, Harhausen et al. [60] identified colocalization between the TSPO-PET tracer [${}^{18}$F]DPA-714 and activated microglia by radiolabeling and immunohistochemical staining.

**Fig. 1:**
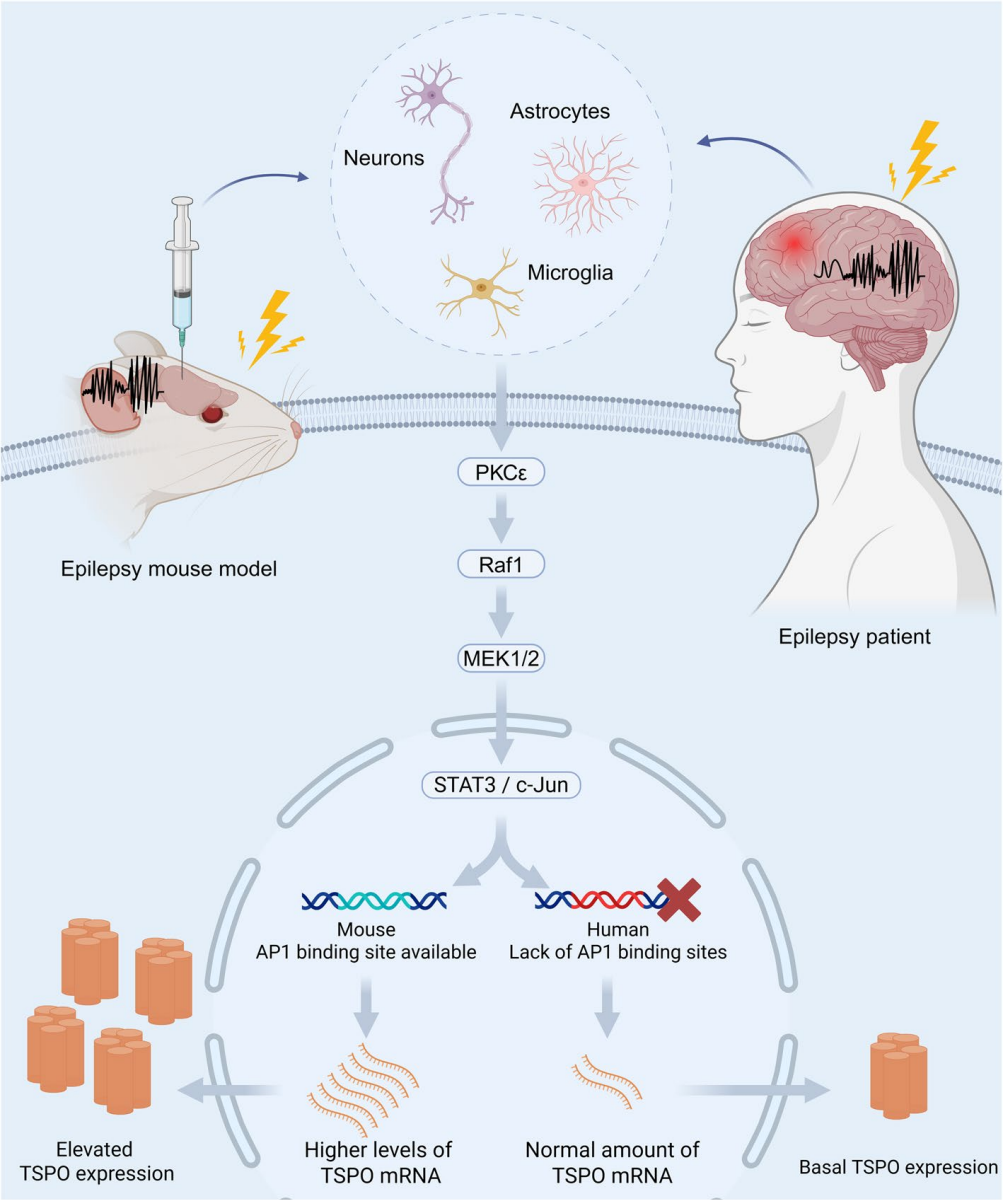
External stimuli like seizures can activate intracellular signaling cascades, leading to the overexpression of the TSPO in microglia and astrocytes within seizure foci. Upon stimulation, the PKC$\epsilon$ pathway is initiated, sequentially activating Raf1, MEK1/2, and ERK1/2. The signaling cascade reaches the nucleus, where transcription factors such as STAT3 and c-Jun combine as AP-1 and modulate gene expression. In mice, the availability of AP-1 binding sites promotes the upregulation of TSPO, enhancing inflammatory responses and mitochondrial dysfunction. However, in humans, the absence of AP-1 binding sites leads to normal TSPO levels under epileptic conditions

While these findings support a well-defined mechanism of TSPO upregulation, primarily established in rodent models, emerging evidence suggests that this pathway may not operate similarly in human microglia. Unlike rodent models, human microglia and monocytes lack activating protein 1 (AP-1) [61], a key factor required for the upregulation of TSPO [62]. Thus, inflammation-induced activation of human microglia does not lead to increased TSPO expression [63, 64] (Fig. 1). Instead, the elevated TSPO signal in seizure foci may result from the accumulation of activated microglia in these regions rather than an upregulation of TSPO at the cellular level.

In addition to microglia, other cell types within seizure foci also express TSPO. For instance, activated astrocytes increase TSPO expression gradually, reaching peak levels between one and six weeks post-seizure, which occurs later than in microglia [23]. Neurons, under certain conditions, may also express small amounts of TSPO [19, 65].

Therefore, increased tracer uptake in TSPO-PET reflects not only the activation and aggregation of microglia but also contributions from astrocytes and, occasionally, neurons. This multifaceted expression of TSPO in pathological conditions enhances the clinical utility of TSPO-PET in identifying seizure foci.

## Advances in TSPO-PET tracer development

**Fig. 2:**
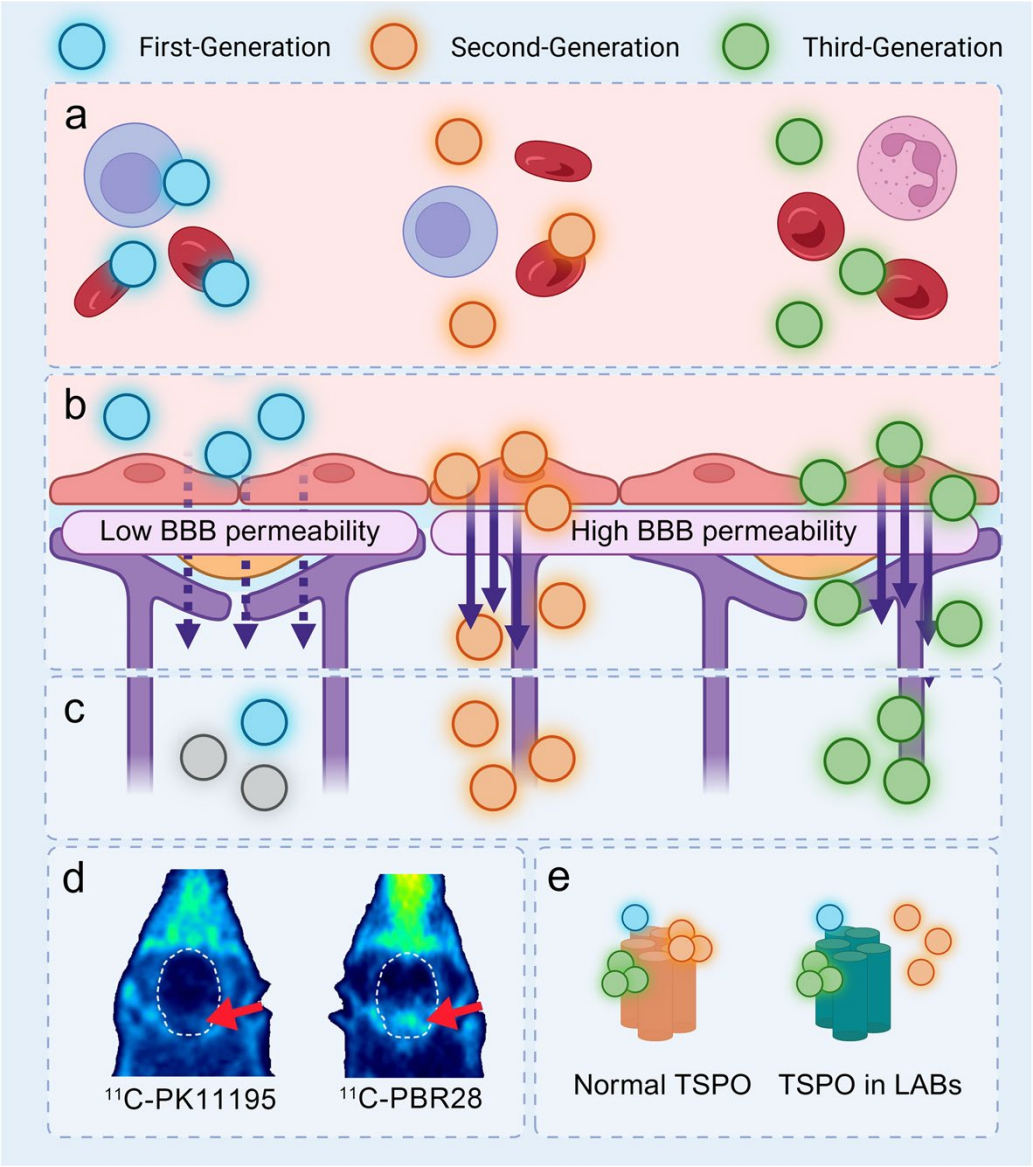
Comparison of TSPO-PET tracers: pharmacokinetics, BBB permeability, binding characteristics, and SNP effects. **a** Blood components and nonspecific binding of TSPO tracers. First-generation (blue) shows high nonspecific binding; second- (orange) and third-generation (green) tracers show minimal peripheral binding. **b** BBB permeability. First-generation tracers show poor penetration, while second- and third-generation tracers cross the BBB efficiently for better brain uptake and contrast. **c** Radiotracer decay. First-generation tracers decay quickly, while second- and third-generation tracers enable longer imaging. **d** TSPO-PET images comparing [${}^{11}$C]PK11195 and [${}^{11}$C]PBR28, showing higher signal-to-noise and contrast with second-generation tracers. Permission was granted by Parente A, et al. (©SNMMI [80]) to reuse this figure. (**e**) rs6971 SNP effects. Second-generation tracers (orange) show reduced binding in LABs; third-generation (green) maintain binding; first-generation (blue) are unaffected but limited by other issues

Over the past three decades, TSPO-PET tracer development has progressed through three generations, each addressing the limitations of its predecessors (Fig. 2).

### First-generation tracers

The first-generation TSPO tracers, [${}^{11}$C]PK11195 [8], has several limitations that restrict its clinical utility, although it has been widely used in both human [9, 66, 67] and animal studies [68, 69] of epilepsy.

First, it exhibits a relatively low signal-to-noise ratio, primarily due to suboptimal blood-brain barrier (BBB) permeability and high nonspecific binding to peripheral components such as blood cells and plasma proteins [70]. Although [${}^{11}$C]PK11195 possesses a favorable lipophilicity (logD = 3.97), this exceeds the optimal range (approximately 2.2 [71]) and contributes to its nonspecific binding [72]. Consequently, its imaging contrast is inferior to that of second- and third-generation tracers, with reported core-to-contralateral uptake ratios of approximately 2.43 ± 0.39 for [${}^{11}$C]PK11195 and 3.41 ± 1.09 for second-generation tracers [73].

Second, as a carbon-11-labeled compound, [${}^{11}$C]PK11195 has a short physical half-life of around 20 minutes, in contrast to fluorine-18-labeled tracers such as [${}^{18}$F]DPA-714, which has a half-life of approximately 110 minutes [72]. This short half-life necessitates the availability of an on-site cyclotron, limiting its use to specialized research centers.

Despite these technical limitations, [${}^{11}$C]PK11195 has been widely applied in both experimental and clinical epilepsy research. In animal models, such as kainic acid-induced status epilepticus, PK11195-PET has successfully visualized neuroinflammatory responses in the hippocampus, amygdala, and medial temporal lobe [74]. In clinical settings, PK11195-PET has been used to detect regional inflammation in patients with refractory epilepsy. For example, Kumar et al. [66] reported that [${}^{11}$C]PK11195-PET was able to reveal seizure foci that were undetectable on MRI and FDG-PET scans.

Although largely replaced by newer tracers in most clinical applications, [${}^{11}$C]PK11195 remains a valuable reference compound-particularly because it is unaffected by the rs6971 single nucleotide polymorphism (SNP) in the *TSPO* gene. This genetic variability can significantly affect the performance of many second-generation tracers, making PK11195 a useful alternative in studies where genotyping is not feasible or when SNP-insensitive tracers are preferred [75].

### Second-generation tracers

Second-generation TSPO tracers, such as [${}^{18}$F]PBR11 and [${}^{11}$C]DPA-713, offer markedly improved signal-to-noise ratios compared to [${}^{11}$C]PK11195. For example, the uptake ratio of second-generation tracers has been reported to be four to six times higher than that of the first-generation tracers [76].

As a result, several second-generation tracers—including [${}^{11}$C]DAC [25], [${}^{11}$C]PBR28 [77–80], [${}^{18}$F]PBR111 [27, 28], [${}^{11}$C]DPA-713 [77, 78], and [${}^{18}$F]DPA-714 [23, 60, 81]—have been widely adopted in both preclinical and clinical epilepsy research.

However, a major limitation of most second-generation tracers is their sensitivity to the rs6971 single nucleotide polymorphism (SNP), which results in an alanine-to-threonine substitution at position 147 in the TSPO protein [82]. This polymorphism influences tracer binding affinity: individuals with the T/T genotype, known as low-affinity binders (LAB), exhibit significantly reduced binding to second-generation tracers [83]. The prevalence of the LAB genotype varies geographically, ranging from 2% to 30% worldwide [82], necessitating genetic screening prior to clinical use. This requirement for genotyping significantly limits the routine clinical application of these tracers.

### Third-generation tracers

Third-generation TSPO tracers, including [${}^{18}$F]GE-38 [70, 84], [${}^{18}$F]LW223 [85], and [${}^{18}$F]BS224 [86],were developed to overcome the limitations of earlier generations. These tracers demonstrate improved sensitivity and higher signal-to-noise ratios compared to first-generation compounds.

Crucially, third-generation tracers are designed to be insensitive to the rs6971 SNP, allowing for reliable imaging across all genotypes without the need for prior genetic screening. For example, the binding affinity ratio between LAB and high-affinity binders (HAB) for third-generation tracers such as [${}^{18}$F]GE-38 and [${}^{18}$F]LW223 ranges from approximately 1.0 to 1.8, which is comparable to the first-generation tracers [${}^{11}$C]PK11195 (approximately 0.8). This represents a significant improvement over second-generation tracers, where the LAB/HAB ratio can range from 4 to 55.3, making third-generation tracers more robust across different genotypes [84].

Demonstrating their practical utility, Russmann et al. [87] used [${}^{18}$F]GE-180, a third-generation TSPO tracer, to perform longitudinal PET imaging in a rat model of temporal lobe epilepsy. They showed that early increases in [${}^{18}$F]GE-180 uptake in specific brain regions, such as the hippocampus and amygdala, could predict the subsequent development of spontaneous seizures, supporting the potential of third-generation TSPO tracers as biomarkers for seizure foci.

Several third-generation tracers are currently undergoing preclinical studies [85] and clinical trials, and their favorable characteristics make them strong candidates to replace earlier generations. Their broader applicability is expected to accelerate the clinical adoption of TSPO-PET imaging.

## Clinical applications of TSPO-PET in preoperative localization of seizure foci in drug-resistant epilepsy

The International League Against Epilepsy recommends that DRE patients undergo a thorough preoperative evaluation using multiple diagnostic tools, including electroencephalography (EEG), MRI, PET, and single-photon emission computed tomography (SPECT) [88]. Among these, MRI, with its high spatial resolution, is the first choice for localizing seizure foci [88]. However, when MRI fails to identify the lesion or the findings do not align with clinical symptoms and EEG results, PET and SPECT can provide additional insights [89]. FDG-PET is often employed to help identify seizure foci in patients with negative MRI results [90].

Despite its utility, FDG-PET has notable limitations. Although FDG-PET is a valuable tool in presurgical evaluation, its localization rate remains limited in patients with MRI-negative epilepsy. Its effectiveness is particularly limited in MRI-negative patients with extratemporal lobe epilepsy, where the positive detection rate of FDG-PET is reported to be only around 30% [91]. Additionally, focal hypometabolism detected by FDG-PET may occur in regions outside the seizure foci [92], which can result in mislocalization [93] and reduced specificity, particularly when combined with MRI in FDG-PET/MRI [94]. Inaccurate identification of seizure foci can result in poor surgical outcomes, adversely affecting the patient’s quality of life [95].

**Fig. 3:**
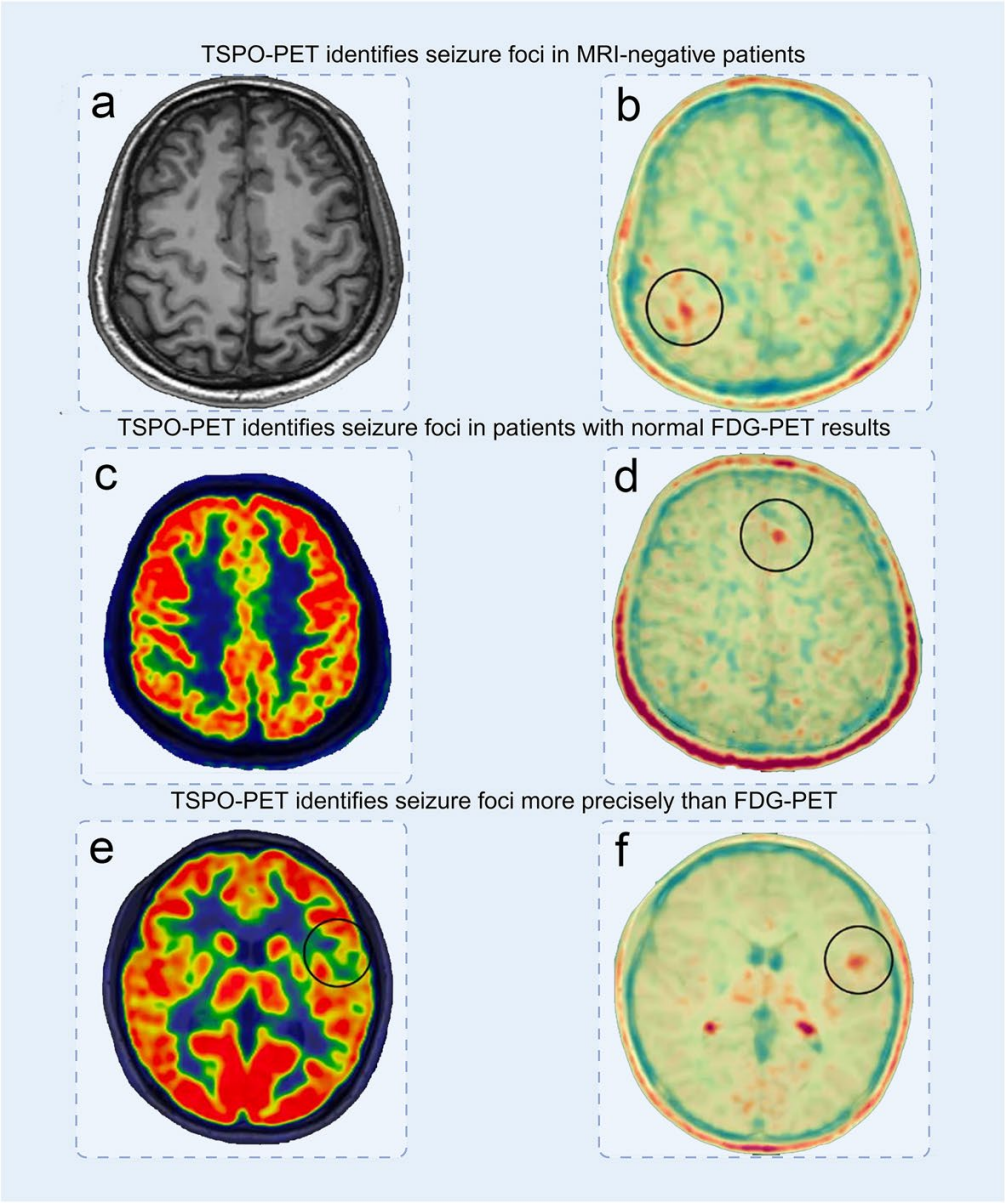
TSPO-PET demonstrates superior seizure focus localization compared to MRI and FDG-PET in various clinical scenarios. **a**, **b** In a patient with normal MRI findings (**a**), TSPO-PET (**b**) reveals a focal area of increased uptake (black circle), indicating an epileptogenic region that was undetectable on structural imaging. **c**-**d** In another case with normal FDG-PET (**c**), TSPO-PET (**d**) identifies a previously unrecognized hyperinflammatory focus (black circle), highlighting its added diagnostic value when metabolic imaging is inconclusive. **e**, **f** In a patient with FDG-PET hypometabolism (**e**), TSPO-PET (**f**) shows more restricted and well-defined uptake (black circle), providing higher spatial precision for surgical planning. Permission was granted by Qiao Z et al. (©Wiley [81] license No. [6015940098201]) to reuse this figure

As a novel imaging modality, TSPO-PET achieves a higher positive detection rate for seizure foci compared to MRI and FDG-PET. Moreover, it can accurately localize the seizure foci in MRI-negative patients (Fig. 3). Therefore, the preoperative use of TSPO-PET for localization is expected to improve patient outcomes (refer Table 1).

**Table 1:** Comparison among TSPO-PET and other imaging outcomes

Study	Category	Sample description	TSPO-PET ligands	TSPO-PET outcomes	Other imaging outcomes
Goerres et al. [96]	Case report	30-year-old woman with a 12-year history of FAS	[${}^{11}$C]-PK11195	Increased regional binding in left occipital, temporal lobes and thalamus	MRI finds atrophy in the left occipital and temporoparietal region
Fujita et al. [97]	Prospective study	9 patients with the diagnosis of neurocysticercosis and history of associated seizures	[${}^{11}$C]-PBR28	A mean of 13% increase in perilesional edema	All patients have structural lesions on MRI
Dickstein et al. [77]	Prospective study	11 patients with DRE	[${}^{11}$C]PBR28 or [${}^{11}$C]DPA-713	81% (9/11) patients have AIs significantly higher than control group in seizure focus. Among FDG-PET negative patients, 71% (5/7) have positive results.	55% (6/11) patients have MRI-positive results. 44% (4/9) TSPO-PET positive patients have MRI-negative results. 22%(2/9) patients have FDG-PET positive results.
Kagitani-Shimono et al. [98]	Prospective study	27 patients with intractable focal epilepsy	[${}^{11}$C]DPA-713	85% (23/27) patients have positive results. 55% (5/9) MRI-negative patients have positive results. 100% (10/10) patients with cortical malformation have positive results	67% (18/27) patients have MRI-positive results. 75% (15/20) patients show focal hypometabolism in FDG-PET results.
Kumar et al. [66]	Case report	5-year-old boy with DRE with normal MRI	[${}^{11}$C]-PK11195	High signal in the left temporo-occipital region	FDG-PET finds severe and diffuse hypometabolism
Butler et al. [67]	Case report	31-year-old woman with DRE with normal MRI	[${}^{11}$C]-PK11195	Focal high signal in the right frontal lobe	FDG-PET finds no clear hypometabolism
Cheval et al. [99]	Case report	17-year-old girl with DRE with normal MRI	[${}^{18}$F]-DPA-714	Focal uptake increase within right precentral area	FDG-PET finds no clear hypometabolism
Cheval et al. [100]	Prospective study	23 patients with DRFE	[${}^{18}$F]-DPA-714	95.7% (22/23) patients have positive results. 100% (15/15) MRI-negative patients have positive results. 100% (13/13) FDG-PET negative patients have positive results in visual analysis, and 70.8% in ROI analysis. In 91.3% (21/23) patients, TSPO-PET reveal more anomalies than FDG-PET	35% (8/23) patients have MRI-positive results. 57% (13/23) patients have FDG-PET positive results. 40%(6/15) MRI-negative patients have FDG-PET positive results
Qiao et al. [81]	Prospective study	24 patients with FCD	[${}^{18}$F]-DPA-714	19 of 24 (79.2%) patients’ results revealed high-level activation. 6 of 11 (54.5%) FDG-PET negative patients have positive results	13 of 24 (54.2%) patients showed positive FDG-PET results
Butler et al. [101]	Case report	35-year-old man with DRE with normal MRI	High signal in bilateral supplementary motor area, the area of abnormality becomes 61% larger after seizure strikes	MRI reveals no significant abnormalities FDG-PET shows left anterior frontal hypometabolism	
Gershen et al. [78]	Prospective study	23 patients with TLE	[${}^{11}$C]DPA-713 and [${}^{11}$C]PBR-28	[${}^{11}$C]DPA-713 finds more focal elevated binding region and higher AIs than [${}^{11}$C]PBR-28	All patients have structural lesions on MRI
Hirvonen et al. [79]	Prospective study	16 patients with TLE	[${}^{11}$C]-PBR28	The hippocampal regions near the seizure focus have increased uptake of radioactivity	69% (11/16) patients have MRI-positive results
Kagitani-Shimono et al. [102]	Cross sectional study	20 patients diagnosed with TSC	[${}^{11}$C]-DPA-713	The volume ratio of [${}^{11}$C]DPA-713-PET/ICV in the no-seizure and refractory seizure groups is negatively correlated with the estimated IQ/DQ	MRI finds cortical tubers and subependymal nodule in all patients
Theodore et al. [103]	Retrospective study	87 patients under surgical treatments (54 with MTS, 22 FCD, 4 tumors, 3 vascular malformations and 3 a history of viral encephalitis)	[${}^{11}$C]-PBR28	Patients infected with HHV-6 has increased binding in seizure focus, while those uninfected don’t	The author did’t mention the results

Abbreviations: *AI* Asymmetry index, *DQ* Developmental quotient, *DRFE* Drug-resistant focal epilepsy, *ETLE* Extra-temporal lobe epilepsy, *FAS* Focal aware seizure, *FCD* Focal cortical dysplasia, *HHV-6* human herpesvirus-6, *ICV* Intracranial volume, *IQ* Intelligence quotient, *MTS* Mesial temporal sclerosis, *ROI* Region of interest, *TSC* Tuberous sclerosis complex

### Higher detection rates of seizure foci with TSPO-PET compared to MRI

TSPO-PET has consistently demonstrated superior detection rates for seizure foci compared to MRI, particularly in challenging cases such as extratemporal epilepsy and MRI-negative patients. Clinical studies have shown that TSPO-PET not only confirms the location of seizure foci in MRI-positive cases [77, 98], but also identifies lesions in patients with normal MRI results, where conventional imaging techniques fail [66, 67, 77, 81, 98, 99].

For instance, Cheval et al. [100] successfully localized seizure foci in all 15 DRE patients with normal MRI findings using TSPO-PET, and these findings were confirmed by surgical outcomes. TSPO-PET revealed signal abnormalities in 100% of the MRI-negative cases, suggesting its potential to overcome one of the key limitations of structural imaging. Similarly, Qiao et al. [81] evaluated a cohort of patients with histopathologically confirmed focal cortical dysplasia (FCD), and found that among 13 patients with MRI-negative findings, TSPO-PET successfully localized the seizure foci in all cases. These localizations were consistent with stereoelectroencephalography and postsurgical histopathological results.

Together, these studies indicate that TSPO-PET can provide additional diagnostic value in MRI-negative epilepsy by revealing inflammation-associated abnormalities that are undetectable with conventional MRI. It also aids in refining the presurgical hypothesis in patients with inconclusive or discordant phase-1 evaluations, thereby improving diagnostic confidence and surgical planning.

Moreover, the combination of TSPO-PET and MRI has been shown to further enhance the detection rate of seizure foci, offering a complementary approach that improves overall diagnostic accuracy. This is particularly valuable in complex cases such as focal cortical dysplasia [81, 104]. While these results establish the utility of TSPO-PET in detecting seizure foci—especially in MRI—negative patients-its performance relative to FDG-PET is further addressed in “Superior detection rates and precision of TSPO-PET compared to FDG-PET” section.

### Superior detection rates and precision of TSPO-PET compared to FDG-PET

TSPO-PET outperforms FDG-PET in both detection rates and precision for localizing seizure foci. Even in cases where FDG-PET fails to detect any abnormality, TSPO-PET has been able to localize seizure foci.

Dickstein et al. [77] investigated 11 patients with DRE, of whom 7 had normal FDG-PET findings. Among these 7 patients, TSPO-PET yielded positive results in 5 cases, as measured by standardized uptake values, asymmetry index, and distribution volume corrected for plasma-free fraction. Of these 5 patients, 3 underwent surgical resection, and all were confirmed to have FCD based on pathological examination—providing strong histological support for the TSPO-PET findings.

In another study, Cheval et al. [100] evaluated 29 patients with DRE, including 15 with normal MRI findings. In cases where FDG-PET failed to identify the seizure foci, TSPO-PET proved highly effective, identifying anomalies in 100% of cases by visual analysis and in 70.8% of cases using region-of-interest (ROI)-based analysis. These findings highlight TSPO-PET’s potential to supplement conventional imaging modalities, particularly in FDG-negative DRE patients.

Moreover, studies indicate that TSPO-PET provides more precise localization than FDG-PET. In the same patient, TSPO-PET signals are typically more restricted to the seizure foci, whereas FDG-PET often shows broader hypometabolic regions on visual analysis, increasing the risk of surgical mislocalization [67, 99]. Cheval et al. [100] further reported that TSPO-PET was more likely to provide new information for refining the localization of the seizure zone (65.2% vs. 17.4% compared to FDG-PET).

However, there are also instances in which high TSPO-PET signals appear outside the actual seizure zone [77, 78, 105], highlighting ongoing challenges with specificity. In patients with TLE, this may be partially attributed to physiological TSPO expression in the adjacent choroid plexus, which can lead to signal spillover and apparent signal enlargement around the hippocampus [78]. In contrast, in non-temporal lobe epilepsy cases, widespread TSPO-PET signals in cortical areas have occasionally been observed, although the underlying mechanisms remain unclear.

## Factors influencing TSPO-PET results

To improve the positive detection rate and accuracy of TSPO-PET for identifying seizure foci, it is essential to analyze the variables that affect imaging outcomes.

**Fig. 4:**
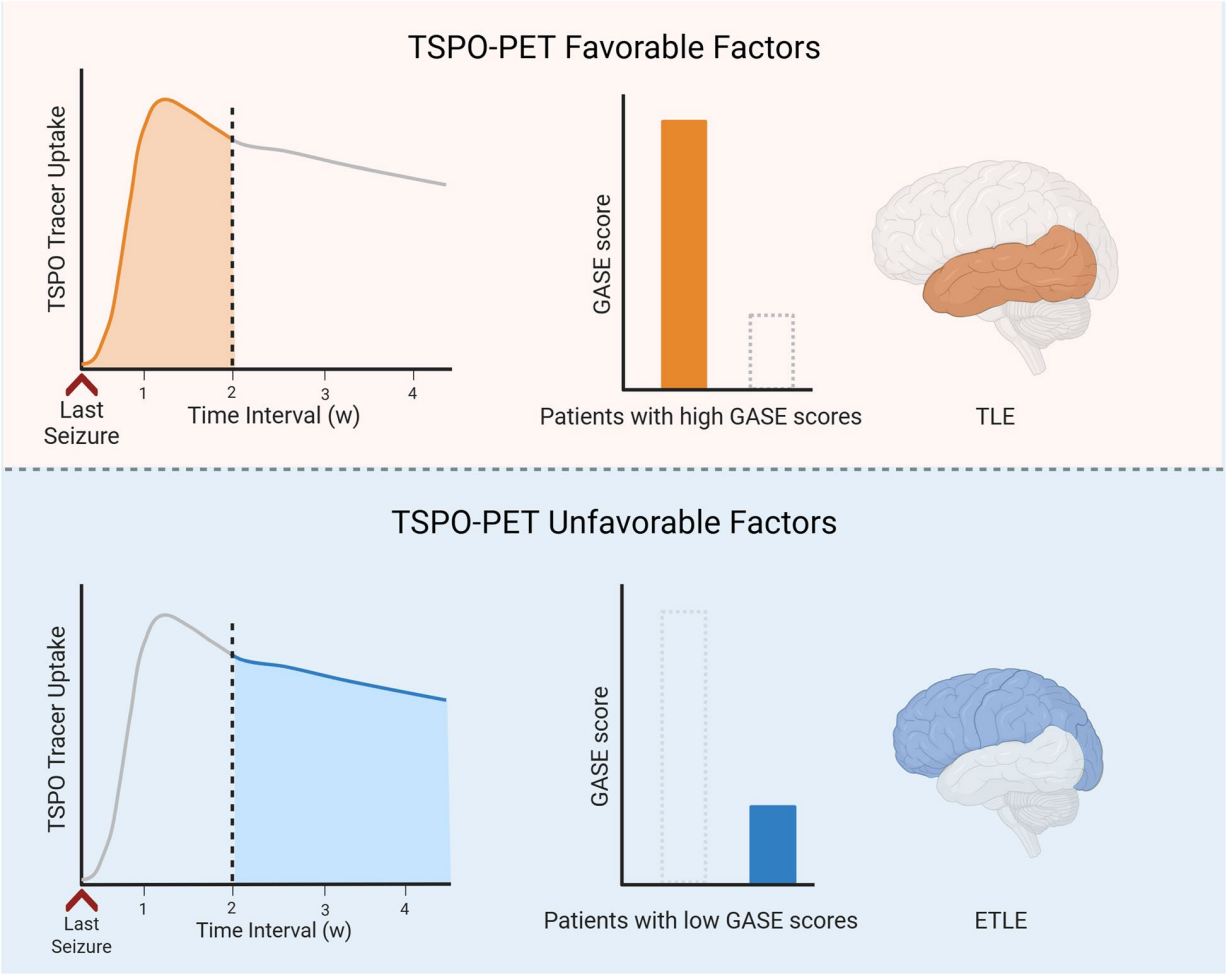
Schematic illustration of favorable and unfavorable factors influencing TSPO-PET imaging results. Key factors affecting TSPO-PET results include the time interval between the last seizure and the TSPO-PET scan—earlier imaging (within approximately 2 weeks) after a seizure is associated with higher TSPO tracer uptake; the severity of epilepsy—patients with higher GASE scores tend to show stronger TSPO-PET signals; the seizure type—patients with TLE tend to show more localized TSPO-PET signals than those with ETLE

Several factors—including the timing of the scan relative to the most recent seizure, epilepsy severity, and other clinical variables such as viral infections [103], as well as findings from FDG-PET or MRI [100]—have been associated with variations in tracer uptake and interpretive reliability (Fig. 4). Among these, the timing of the scan and the severity of epilepsy are the most consistently studied and will be the primary focus of this section.

### Time interval between the last seizure and TSPO-PET scan

The interval between a patient’s most recent seizure and the TSPO-PET scan can impact the tracer uptake in the seizure focus. Butler et al. [101] demonstrated that tracer uptake was higher when the TSPO-PET scan was performed shortly after a seizure compared to the interictal period.

To determine the optimal timing for TSPO-PET, researchers have tracked the temporal changes in tracer uptake following seizures. In most brain regions, tracer uptake begins to increase within 5 to 10 days after the first seizure, peaking at approximately one week [68, 106]. Regions particularly susceptible to seizures, such as the hippocampus, exhibit the earliest and most pronounced tracer uptake [107]. Over the subsequent 4 to 12 weeks, uptake levels gradually declines, though the seizure focus and ipsilateral hippocampus tend to retain elevated uptake levels for longer [68, 105, 106].

These dynamics are thought to reflect underlying biological processes such as microglial activation, which typically peaks around one week post-seizure [68], and transient BBB disruption [108], which increases permeability to TSPO tracers. Notably, such BBB compromise may also contribute to non-specific or misleading uptake patterns, especially during the acute postictal period [77, 78, 105].

While elevated TSPO-PET signals shortly after seizures may reflect true neuroinflammatory activity, they should not be interpreted as false positives per se. Rather, they emphasize the importance of careful image interpretation in the context of clinical timing. Transient, non-localized uptake-particularly in perilesional or contralateral regions-has been observed in both animal models and clinical cases [77, 78, 105], and may result from diffuse inflammatory responses or temporary BBB leakage. Thus, integrating electroclinical data is critical for accurate interpretation. Further studies are needed to better define the prevalence and diagnostic implications of such non-specific signal patterns.

Based on the current evidence, the optimal time window for TSPO-PET acquisition is approximately one week following the last seizure, during which tracer uptake is most reliably correlated with the seizure foci.

### Severity of epilepsy

Epilepsy severity is another key factor influencing TSPO tracer uptake. Kagitani-Shimono et al. [102] used the Global Assessment of Severity of Epilepsy (GASE) scale to assess epilepsy severity and found that patients with higher GASE scores exhibited larger areas of high TSPO uptake. Other indicators of epilepsy severity, such as seizure frequency [102] and hippocampal sclerosis on the ipsilateral side of the focus [79], were also associated with higher tracer uptake in the seizure foci.

### Others

Beyond seizure timing and severity, several additional variables may affect TSPO-PET results. For example, seizure type could influence uptake patterns: although all are focal seizures, TLE tends to produce more localized signals, while ETLE may generate more widespread or diffuse uptake patterns, complicating the delineation of the seizure focus [77]. Although the specific impact of antiseizure medications (ASMs) on TSPO-PET imaging remains unclear, several potential mechanisms have been hypothesized. Some ASMs, particularly those with anti-inflammatory properties (e.g., valproate [109]), may theoretically suppress glial activation and thus reduce TSPO expression. However, no systematic clinical studies have been conducted to directly evaluate the influence of ASMs on TSPO-PET signals in epilepsy. Animal studies investigating ASM effects on TSPO expression could help to establish a foundation for future research. Currently, evidence supporting these hypotheses is limited, underscoring the need for future research.

## Limitations of TSPO-PET in epilepsy evaluation

Despite growing interest in TSPO-PET for the presurgical evaluation of DRE, several limitations constrain its current clinical utility. First, although third-generation tracers, such as [${}^{18}$F]GE-180 [105] and [${}^{18}$F]BS224 [86], have overcome the issue of binding affinity variability caused by the rs6971 polymorphism, TSPO-PET still yields negative or misleading results in a subset of patients. One key issue is that TSPO-PET reflects neuroinflammation, which is not necessarily specific to seizure foci. Regions of increased TSPO uptake may also arise from non-epileptic pathology such as trauma, gliosis, or neurodegeneration, potentially leading to false positives. Furthermore, the TSPO signal is not always strictly colocalized with seizure onset zones, complicating clinical interpretation. Another important limitation is the lack of cell-type specificity: TSPO is upregulated in various activated glial and immune cells, including microglia, astrocytes, and peripheral macrophages, and the relative contribution of these populations to the PET signal in epilepsy remains unclear. These issues limit the precision and reliability of TSPO-PET for localizing seizure foci in clinical practice.

## Future research directions

Looking ahead, several strategies may enhance the clinical value of TSPO-PET for seizure focus localization. Multimodal imaging approaches, particularly combinations with MRI, FDG-PET, or potentially SPECT, could improve accuracy by integrating complementary structural, metabolic, and perfusion-related information [109]. Standardizing image acquisition protocols, quantification methods, and interpretation criteria will be essential for broader clinical adoption. Future research should also clarify the cellular and molecular sources of TSPO signals within seizure foci. Techniques such as single-cell transcriptomics and spatial mapping could help identify which cell types dominate the PET signal in epileptic tissue. Additionally, investigating how inflammatory responses evolve within seizure foci over time may refine the timing and interpretation of TSPO-PET scans. By advancing both technical methods and biological understanding, TSPO-PET has the potential to become a more reliable tool for seizure focus localization in DRE.

## Conclusion

TSPO-PET is a promising imaging modality that can assist clinicians in the preoperative localization of seizure foci in DRE. By targeting the overexpression of TSPO in activated microglia and other glial cells, TSPO-PET tracers offer valuable insights into inflammatory processes associated with epilepsy.

Compared to conventional modalities such as MRI and FDG-PET, TSPO-PET demonstrates superior localization performance in several clinical scenarios. It has been shown to identify epileptogenic foci in MRI-negative patients and extratemporal epilepsy, where FDG-PET often shows low sensitivity or diffuse uptake patterns. In addition, the spatial precision of TSPO-PET can reduce the risk of surgical mislocalization, thereby improving postoperative outcomes. When combined with MRI, TSPO-PET further enhances the detection rate and diagnostic confidence, particularly in patients with complex or ambiguous presentations.

Nonetheless, TSPO-PET is not without limitations. Its signal may be influenced by seizure timing, disease severity, and-depending on the tracer-genetic polymorphisms such as rs6971. Moreover, TSPO expression is not exclusive to the seizure foci, and transient inflammatory changes may confound interpretation. These challenges highlight the need for standardized acquisition protocols, careful clinical correlation, and continued research into the cellular and molecular determinants of TSPO signal.

With the ongoing development of third-generation tracers and the increasing availability of multimodal imaging platforms, TSPO-PET is well-positioned to become a valuable addition to the clinical toolbox for DRE. Its integration into comprehensive presurgical evaluation has the potential to refine focus localization, guide resection strategy, and ultimately improve patient outcomes.

## Data Availability

Not applicable.

## Abbreviations

AI: Asymmetry index
AP-1: Activating protein 1
BBB: Blood-brain barrier
CRAC: Cholesterol recognition/interaction amino acid consensus
DRE: Drug-resistant epilepsy
EEG: Electroencephalography
ETLE: Extra-temporal lobe epilepsy
FAS: Focal aware seizure
FCD: Focal cortical dysplasia
FDG: Fluorodeoxyglucose
GASE: Global assessment of severity of epilepsy
ICV: Intracranial volume
ILAE: International League Against Epilepsy
IQ: Intelligence quotient
LAB: Low-affinity binders
M0: Resting microglia
M1: Pro-inflammatory microglia
M2: Anti-inflammatory microglia
MRI: Magnetic resonance imaging
mPTP: Mitochondrial permeability transition pore
MTS: Mesial temporal sclerosis
NOX2: NADPH oxidase complex
PET: Positron emission tomography
PKC$\epsilon$: Protein kinase C epsilon
ROS: Reactive oxygen species
SNP: Single nucleotide polymorphism
SPECT: Single-photon emission computed tomography
SQSTM1: Sequestosome 1
TLE: Temporal lobe epilepsy
TSC: Tuberous sclerosis complex
TSPO: Translocator protein
VDAC1: Voltage-dependent anion channel 1
